# Factors That Influence Burnout of Clinical and Research Faculty: New Insights of Data from a United States Cancer Center Using CART Analysis

**DOI:** 10.3390/healthcare14070926

**Published:** 2026-04-02

**Authors:** Shine Chang, Hwa Young Lee, Katelyn J. Cavanaugh, Courtney L. Holladay

**Affiliations:** 1 Cancer Prevention Research Training Program, Division of Cancer Prevention and Population Sciences, The University of Texas MD Anderson Cancer Center, Houston, TX 77030, USA; hlee12@mdanderson.org; 2 Department of Health Disparities Research, Division of Cancer Prevention and Population Sciences, The University of Texas MD Anderson Cancer Center, Houston, TX 77230, USA; 3 Leadership Institute, Division of Human Resources, The University of Texas MD Anderson Cancer Center, Houston, TX 77030, USA; kjcavanaugh@mdanderson.org (K.J.C.); clholladay@mdanderson.org (C.L.H.)

**Keywords:** faculty burnout, health professionals, classification and regression tree (CART), institutional support, leadership

## Abstract

**Highlights:**

**What are the main findings?**

- In analysis of data from faculty at a U.S. academic cancer center employed in both 2019 and 2021, specific aspects of faculty engagement with the institution, its leadership, and faculty perceptions of work–life quality and control over their work were associated with report of burnout in 2021, revealing opportunities for institutional intervention.
- While prior burnout was predominantly associated with future burnout, it alone did not doom all faculty to future burnout as other factors were also independently associated with future burnout, including for those not reporting burnout earlier.

**What are the implications of the main findings?**

- Individual-level interventions to prevent and ameliorate burnout are important as others report, but our work revealed that faculty perception and experience of institutional-level factors may also be key to addressing faculty burnout at similar academic health science centers.
- Strategies to promote inclusive institutional climate and autonomy over work may play critical roles in preventing and managing risk of faculty burnout at academic health science centers.

**Abstract:**

**Background/Objectives:** Burnout among academic health professionals affects well-being and performance of critical responsibilities—clinical, research, administrative, and teaching. Despite growing attention, study limitations hinder understanding the mechanisms of burnout among health professionals fully. This study identifies individual and institutional factors associated with faculty burnout at a U.S. academic cancer center. **Methods:** From 2019 to 2021, all faculty at a large research hospital, regardless of rank, were invited to complete employee surveys, which assessed institutional support, work–life balance, and job demands. Burnout in 2021 served as the primary outcome, measured using a validated single-item scale with five response options: 1–2 were classified as “not burned out” and 3–5 as “burned out.” Using classification and regression tree (CART) analysis, a flexible, non-parametric approach that does not require distributional assumptions of the outcome variable and is well-suited for handling complex, non-linear relationships and interactions among multiple predictors, we explored without a priori hypotheses factors contributing to burnout status in 2021, using prior burnout experience and institutional factors assessed in both years as predictors. **Results:** This cross-sectional analysis revealed both report of burnout in 2019 and perceptions of low institutional inclusion linked to burnout in 2021, while higher report of job accomplishment and of empowerment was associated with lower burnout in 2021. Past burnout did not doom faculty to future burnout when they felt a strong sense of institutional inclusion and support in adapting to institutional change, indicating that burnout can be mitigated, even after a pandemic. **Conclusions:** Patterns of burnout were related to faculty engagement with the institution and leadership and their perceptions of work–life quality and control over their work, revealing opportunities for intervention. Strengthening support systems, promoting strategies for managing professional and personal demands better, and optimizing workloads may mitigate risk for faculty in academic health centers.

## 1. Introduction

Interest in burnout has grown in all sectors, particularly in health care given the role of professionals in preserving and restoring the health of others. Major efforts focus on measuring and understanding burnout among clinical professionals and others at academic health centers across the country. These include estimating the prevalence of burnout [1], comparisons of burnout reported by clinicians and researchers [2,3] and across gender [4], the negative correlates of burnout on both individuals experiencing burnout and outcomes such as patient care [5], and the interventions to reduce burnout [6]. These studies have informed and raised concern appropriately, but methodological limitations constrain greater understanding of burnout affecting health care and research professionals.

A key limitation to previous research on burnout is in study design. Cross-sectional (i.e., risk factors and outcomes collected at a single time point) and ecologic (i.e., risk factors attributable to groups, not individuals) designs [7,8] by nature do not constitute reliable measures of burnout incidence over time nor can they link specific factors or interventions causally to changes in rates of burnout. In response, several teams have employed longitudinal designs and advanced statistical analyses such as latent profile and latent growth curve modelling to investigate nurse [9] and healthcare worker [10,11] burnout levels over time. However, even such studies employing longitudinal design have shortcomings, such as low response rate [12] and focus on trainees, which limit the generalizability of their findings. Other limitations stem from the methods used to aggregate professionals from different types of institutions and omission of institutional features as factors that may moderate the experience of burnout among healthcare professionals. Many investigations focus on a single type of healthcare professional, omitting the burnout experience of other professional disciplines within the same healthcare institutions [13,14,15]. Also, few studies include institutional features, although in one cross-sectional survey of 65 assistant professors within a single pediatrics department, three institutional factors were related to lower burnout: feeling departmental support for well-being, feeling valued for department contributions, and feeling empowered to communicate professional development needs [8]. These limitations make urgent additional research on the drivers of burnout among academic faculty [16].

We describe the burnout experience of a sizable and diverse faculty at a single large healthcare and research institution in the United States over a two-year period (i.e., 2019 and 2021). For this cross-sectional analysis, we took advantage of data from a biennial, confidential employee survey designed for understanding and improving employee engagement. Since few studies have examined institutional factors affecting faculty burnout, importantly described in theoretical frameworks such as the Job Demands-Resources model [17], it is unknown how and which institutional factors are associated with burnout. To address this methodological gap, we used a classification and regression tree (CART) analysis to explore factors contributing to burnout status in 2021 without a priori hypotheses, using prior burnout experience and institutional factors assessed in both years as “predictors.” As a flexible, non-parametric approach that does not require distributional assumptions of the outcome variable [18], CART is well-suited for handling complex, non-linear relationships and interactions among multiple predictors, and avoiding multicollinearity in traditional regression models [19].

## 2. Materials and Methods

### 2.1. Survey Administration

Administered by an external survey consulting company to ensure respondent confidentiality, a biennial survey conducted by The University of Texas MD Anderson Cancer Center, a large cancer-focused academic health care and research institution in Houston, Texas, United States, was used to collect data to assess employee satisfaction and identify factors that influence performance. Surveys were open for two weeks in February 2019 and in February 2021 and overall staff response rates were 89% and 91%, respectively. All faculty employed by the institution for at least one month prior to the survey opening were invited to complete the survey. Significant institutional messaging from leaders at all levels and in multiple settings about the goals and purpose of the survey helped achieve high response rates. The survey contained multiple-choice items designed to assess job and organizational attitudes. Demographic characteristics of faculty including rank (Instructor/Assistant, Associate, Full Professor), gender (female, male), race, years of service at the institution, birth cohort generations (Traditionalist = 1928–1945; Boomer = 1946–1964; Gen X = 1965–1980; Millennial = 1981–1996), and department type (clinical vs. research) were provided to the survey company to coordinate surveys.

#### Study Population

Of 1,788 faculty employed in 2019 and invited to complete the 2019 survey, 1,460 responded (82%). In 2021, 1,656 of 1,830 faculty responded to the survey, yielding a response rate of 90.5%. Among the 1,221 faculty who completed surveys in both years, 46 were excluded because they did not provide a burnout response in the 2019 survey and 24 for not providing a burnout response in 2021. Also, 19 medical physicists and senior medical physicists were excluded. Thus, data for this analysis were available from 1,132 individual faculty. Nearly 40% were women (*n* = 449, Table 1) and large proportions identified as White (49%, *n* = 555) or Asian (40%, *n* = 453). More individuals were Instructor/Assistant Professors (31%, *n* = 353) than Associate (28%, *n* = 315) or Full Professors (30%, *n* = 342).

### 2.2. Measures

Many items and instruments within the survey have been validated in other samples [20] and are used by many organizations for benchmarking purposes. Each year, an institutional panel of internal assessment experts (including one of the authors, CLH), with specialized training in survey design and no conflicts of interest, reviews and chooses survey items for biennial reporting and comparison of changes over time. The survey is refined to minimize respondent burden while optimizing the number of measured constructs important for ongoing or new business needs and organizational goals established by institutional leadership. This review is done in concert with the survey vendor to ensure items remain valid and robust for benchmarking based on their experience within and across many organizations.

#### 2.2.1. Burnout

Burnout in both surveys was measured with a single item [21]: “Overall, based on your definition of burnout, how would you rate your level of burnout?” Scores ranged from 1 to 5, with the following options: (1) “I enjoy my work. I have no symptoms of burnout.”; (2) “Occasionally I am under stress, and I don’t always have as much energy as I once did, but I don’t feel burned out.”; (3) “I am definitely burning out and have one or more symptoms of burnout, such as physical and emotional exhaustion.”; (4) “The symptoms of burnout that I’m experiencing won’t go away. I think about frustration at work a lot.”; and (5) “I feel completely burned out and often wonder if I can go on. I am at the point where I may need some changes or may need to seek some sort of help.” Following the standard cut-point employed in other studies for this burnout item [20,21,22], individuals were categorized into two groups with those reporting burnout scores of 1 and 2 as “not burned out” and those reporting scores of 3 and above as “burned out.” The outcome variable for analysis was burnout status reported in 2021.

#### 2.2.2. Job Attitudes

Job and organizational attitude items were assessed in both years. In 2019, these were measured using 38 multiple-choice items, each with 5-point Likert-type scale responses (1 = Strongly Disagree to 5 = Strongly Agree). These items were used to create constructs for eight workforce dimensions from our previous work [20]: Agility, Alignment, Development, Leadership, Resources, Respect and Trust (measuring respect, trust, inclusion, and diversity), Safety, and Teamwork. For each domain, scale scores were calculated by averaging responses to items associated with each domain. The internal consistency (Cronbach’s alpha) was calculated for each domain that included multiple items but not when a domain consisted of a single item (see Appendix A). The reliability coefficients for each construct, indicating the consistency of multiple items to describe a single construct, ranged from 0.69 (Teamwork) to 0.89 (Leadership).

The panel of internal assessment experts evaluated item relevance, clarity, and alignment with intended domains. For the 2021 survey, three domains from the 2019 survey were excluded (i.e., Agility, Attitude, Belongingness) and six new domains were added (see Appendix A). By analyzing the predictors of burnout for each year independently, not as repeated measures, our primary goal was to ensure that measurements best reflected the state of each domain at that specific point in time. The 2021 revisions improved content validity by incorporating more precise indicators of each domain that were aligned with the evolving institutional priorities and the specific context of that year. See Appendix A for a table of all survey categories, reliability coefficients, and example items from the 2019 and 2021 surveys.

#### 2.2.3. Demographic Characteristics

Demographic variables in the analysis included academic rank, department type, years of service at the institution, gender, race, and birth cohort generations. Although these were assessed in both years, those data from the 2019 survey were used for our analysis. Sixteen Traditionalist faculty (1928–1945) were combined with 367 in the Boomer group.

### 2.3. Implementation of the Classification and Regression Tree (CART) Algorithm

Before conducting the analyses, we performed Little’s MCAR test [23] to assess whether missing data for burnout were Missing Completely at Random (MCAR) using sex, race, generation, job rank, years of service at the institution, and department type as auxiliary variables. The result indicated that missing data for burnout were not related to any demographic variables (*χ*^2^ = 0.28, *df* = 1, *p* = 0.867).

For the main analysis, we used Classification and Regression Tree (CART) to identify factors contributing to or reducing burnout in 2021. The 2021 burnout outcome variable was binary (1 = burnout, 0 = not burnout). Predictors included burnout in 2019 and faculty demographic characteristics along with job and organizational attitude measures assessed in both years.

Survey data were randomly divided into training and testing datasets, for which 70% of the data formed the training dataset and 30% the testing. To ensure reproducibility of the random split, we set a random seed. The training dataset was used to train machine learning models, and the testing dataset was used to verify the classification accuracy of the CART results derived from the training analysis. Models were fit in R (version 4.4.1) with R Studio (version 2024.09.1) [24] using “rpart (Version 4.1-3).” [25] CART used the Gini impurity criterion by default to determine the optimal splits for the classification tree. To control the complexity of the decision tree, we used various complexity parameter (CP) values, ranging from 0.1 to 0.008 because the optimal CP value can vary depending on the specific datasets and fields.

To test classification accuracy of the CART results, four statistics were used: Accuracy, Sensitivity, Specificity, and Positive Predictive Value (PPV; often called “Precision” in machine learning literature). Accuracy measures a model’s overall performance, indicating the proportion of all correct predictions (true positive and true negatives) out of the total number of predictions made. Sensitivity is the proportion of true positives of all cases that are truly positive, representing the model’s ability to correctly identify positive cases (and avoid false negatives). Specificity is the ratio of true negative cases of all those truly negative, reflecting the model’s ability to correctly identify negative cases (and avoid false positives). PPV is the ratio of true positives of the sum of true positive and false positives, indicating how correctly the model identifies positive cases.

## 3. Results

The CART analysis was performed using a complexity parameter (CP) of 0.008 to optimize model performance and to capture meaningful interactions among significant predictors of the outcomes. Model performance was evaluated using 10-fold cross-validation, which is the standard procedure in the rpart package in R, to assess the stability of the final tree. The model demonstrated reasonably strong accuracy of prediction (0.79, Table 2). Model sensitivity to identify actual cases of burnout in 2021 correctly was 62% and specificity to indicate individuals not burned out in 2021 correctly was 88%, highlighting the model’s stronger ability to avoid false positives. For PPV, the model correctly predicted 2021 burnout in 72% of faculty reporting burnout in 2021.

Accuracy, (TP + TN)/(TP + TN + FP + FN): 0.79
Sensitivity, TP/(TP + FN): 0.62
Specificity, TN/(TN + FP): 0.88
Precision, TP/(TP + FP): 0.72

The CART analysis retained all predictors that contributed at least 1% of the total reduction in Gini impurity, providing a comprehensive view of the variables that influenced the model’s performance (see Table 3 for importance ranking). Variable importance was dominated by previous burnout in 2019 (100% relative importance), which served as the first node and the most significant predictor of the outcome. The predictors, job accomplishment in 2021 and inclusion in 2021, also emerged as the important predictors.

Several predictors with relatively high importance ranking, regardless of whether they were assessed in 2019 or 2021 from 2019, did not appear in the visual tree structure. While variable importance captures a predictor’s overall contribution to reducing Gini impurity across all possible splits, the final tree represents a parsimonious subset of splits selected based on the model complexity. When predictors share substantial variance or are highly correlated, the tree typically selects one representative variable for splitting, indicating that the absence of certain high-ranking predictors from the tree reflects overlapping information shared among variables rather than a lack of importance.

### 3.1. Predictors of Burnout

**Figure 1:**
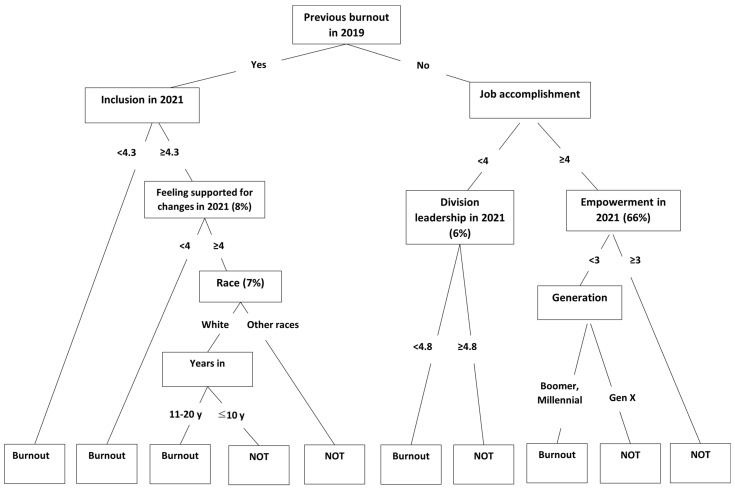
CART Decision Tree of Burnout Predictors.

Many variables were tested using the CART model for contribution to the decision tree (see Appendix A), but here, only the statistically significant associations with burnout status in 2021 are described. The proportion of faculty reporting burnout in the training dataset was 29% in 2019 and 33% in 2021. The CART analysis identified and ranked factors contributing to faculty burnout in 2021, revealing the most powerful driver was a report of burnout in 2019 (Figure 1). Among those reporting burnout in 2019, the next most important factor was their sense of institutional inclusion in 2021, where those with a lower sense of institutional inclusion in 2021 (<4.3) were much more likely to report burnout in 2021; together, these two variables explained the largest group reporting burnout in 2021 (21%). Burnout in 2021 was further associated with perceptions in 2021 of support in adapting to institutional changes. For those reporting burnout in 2019 but having a stronger sense of institutional inclusion in 2021 (≥4.3), faculty burnout in 2021 for 1% of faculty was associated with perceptions of having relatively low support in adapting to institutional changes in 2021. Faculty race and work history also had impact on 2021 burnout; among those reporting burnout in 2019 who had stronger feelings of institutional inclusion (≥4.3) and higher support in adapting to institutional changes (≥4.0), being White and having worked longer at MD Anderson (11–20 y) was associated with burnout in 2021—for 2% of all faculty in 2021.

### 3.2. Predictors of Not Reporting Burnout in 2021

Our analysis also yielded information about factors not predicting burnout; in 2021, approximately 71% of the faculty in the training dataset reported not feeling burned out. Unsurprisingly, the strongest predictor of no burnout in 2021 was not having reported burnout in 2019; of this group, those reporting in 2021 having both higher levels (≥4) of Engagement, the construct that included assessed feelings about job accomplishments, and at least a moderate level (≥3) of Respect and Trust, the construct in which control over work was assessed, were more likely not to report burnout in 2021, the largest group of faculty to do so (63%). Interestingly, others who also reported no burnout in 2019 and higher levels (≥4) of Engagement, but lower levels of Respect and Trust (<3), and membership in Boomer or Millennial generations were more likely to report burnout in 2021 (1%), suggesting the importance of longevity or, conversely, early career enthusiasm to withstand burnout. Still, others not reporting burnout in 2019 who had both relatively low Engagement in 2021 (<4) and relatively low satisfaction with Divisional Leadership (<4.8) reported burnout in 2021 (5%); but those similar except for having higher satisfaction with their Divisional Leadership did not report 2021 burnout (1%), implying the potential for key impact for interventions at the level of divisional leadership.

## 4. Discussion

In this CART analysis of burnout among faculty at a U.S. academic health center, our findings both echo observations from prior studies [20,26] and reveal new directions for empirical exploration of the drivers of institutional workforce burnout, laying the foundation for designing effective organizational interventions. Specifically, prior experience of burnout was the strongest variable associated with faculty burnout in 2021, highlighting the state-dependent nature of burnout across years. Including prior burnout as a variable in analysis allows baseline accommodation that better permits the assessment of other factors being evaluated together, especially when one factor, such as prior burnout, is especially influential. Thus, other factors that contribute to the outcome (i.e., burnout in 2021) beyond an individual’s history can be revealed. However, a potential limitation is that unresolved, persistent burnout reported in 2019 also manifests as lower feelings of inclusion and feeling unsupported during institutional changes, such that these are concurrent to burnout in 2021 and not driving it, potentially even being a result of it. Notably, those with a lower sense of institutional inclusion in 2021 who had a history of burnout in 2019 accounted for the largest group in this analysis reporting burnout in 2021 (21%), more than one fifth of all faculty. Other contributing factors included lower support for adapting to institutional changes and work history, highlighting the nuanced interplay between past burnout experiences and current institutional dynamics.

Conversely, the absence of burnout earlier, coupled with higher job accomplishment and a moderate sense of empowerment, emerged as the strongest factors related to faculty not experiencing burnout in 2021. Identification of these constructs as relevant to lower likelihood of burnout is important as we speculate that they indirectly suggest that faculty control over their work and the satisfaction derived from it may be key for burnout prevention. Similar findings have also been reported by others [27,28] and posited by models of burnout such as the Job Demand—Resources model [17], focusing attention on institutional practices giving faculty discretion over their work. However, even among those without prior burnout, lower job accomplishment and dissatisfaction with leadership predicted burnout in 2021, indicating that burnout risk is not mollified by lack of previous burnout. One can speculate that these factors may be important harbingers to future sustained burnout among faculty in academic health settings.

As we did, others have reported significant associations between clinician perception of leadership attributes and burnout [29,30]. By identifying what drives and mitigates burnout over time, our study extends the literature, offering actionable insights for academic health institutions striving to cultivate healthier and more inclusive work environments. Specifically, some faculty—even though reporting previous burnout—did not report burnout later when they felt a strong sense of institutional inclusion and strong support in adapting to institutional change, indicating that prior report of burnout may be mitigated, even after surviving a global pandemic, which is a key finding. That said, collecting detailed data about individual-level factors in the context of environmental drivers related to burnout can be critical to informing the results of organizational analyses and the development of multi-level interventions to prevent and ameliorate burnout. Understanding whether suffering burnout episodically or continuously over time, or how other individual factors such as coping style or personality, data which were not available for this analysis, affect risk or recovery from burnout or require different approaches for intervention have key implications. Designing interventions that address critical factors influencing faculty burnout must be carefully evaluated for efficacy as evidence accumulates for increases in physician burnout and report from individual physicians of stable burnout status measured over time [12].

This analysis had other limitations and strengths. For one, data were collected at one institution with a single disease focus, limiting generalizability to organizations different in disease focus, structure, and employee composition. That said, aspects of the survey administration, such as a high response rate and data on a variety of topics relevant to employee experience, were strengths. In particular, data available at two time points for a high proportion of eligible faculty allowed powerful analysis of individual-, environmental-, and institutional-level factors associated with burnout in 2021 after accounting for experience of earlier burnout. Multi-level analysis, beyond that of the individual, is also unique, particularly with such a large sample. To our knowledge, few large-scale analyses have examined burnout over a two-year period among academic healthcare faculty, and none at a cancer center. Because this study occurred during the COVID-19 pandemic—a period associated with increased burnout [31]—examining factors related to burnout was especially important. However, our aim was not to determine whether burnout increased or decreased, but to identify variables associated with it. Pandemic-related disruptions and institutional responses to mitigate likely influenced faculty reports of burnout, although these factors were not directly measured.

Because the study included only faculty who responded to the survey at both time points, individuals who did not respond or who left the institution—potentially those experiencing higher levels of burnout—may be underrepresented. For example, whether factors influenced burnout report differently for Traditionalists who may be nearing retirement than for Boomers is unknown, as is a potential effect upon our CART analysis results; however, two sensitivity analyses showed that the overall model structure and performance remained stable, although some lower-level nodes were replaced by alternate predictors that had not appeared in the original model. This pattern may reflect a known limitation of CART analysis, in which lower-level splits can be unstable due to small subgroup sizes or intercorrelations among several predictors. Further studies could apply Random Forest methods to identify the important predictors with more stability. Regardless, this population warrants further investigation, and future work should explore strategies to identify and support individuals at risk before disengagement or departure. Future research should also assess whether the same patterns of associated factors persist in the post-pandemic period.

Nevertheless, CART analysis examining factors associated with individual burnout status by ranked strength is a powerful strategy allowing the data to reveal what drives the patterns. Here, our results demonstrated relatively strong overall model performance, indicating the model’s effectiveness in correctly identifying non-burnout cases while conservatively implicating factors associated with burnout. CART is also better for dealing with large numbers of variables, compared to traditional regression analysis where multicollinearity is a threat. Furthermore, to our knowledge, no study has examined faculty burnout using such a method.

Including prior burnout as a variable in analysis has advantages and disadvantages. One advantage is that managing the influence of burnout at baseline increases the precision of the model, similar to ANCOVA or a lagged-outcome model, by allowing examination of how other variables assessed in both years are also associated with the burnout outcome after accounting for prior burnout. Indeed, our results indicate that even after accommodating prior burnout, other variables were associated independently with future burnout. At the same time, the prior burnout variable (burnout in 2019) may capture some variance shared with other factors assessed in 2019, so their associations with burnout in 2021 may be attenuated when prior burnout in 2019 is included in models. Also, because job and organizational attitudes were assessed at the same time as the 2021 burnout outcome, we cannot infer the direction of influence between them and burnout in 2021. Although the overall accuracy, specificity, and precision indicate general good performance, the model’s relatively lower sensitivity (0.62) to correctly identify burnout cases is a limitation. This hints that predicting burnout is more complex than predicting non-burnout cases due to the multifactorial causes of burnout and the likely heterogeneity of those reporting burnout. As a result, the model, in spite of demographic characteristics and job and organizational attitude measures, did not capture all the contributors to burnout. However, to our knowledge, because this is the first use of CART analysis to classify and rank contributors to burnout, no comparable studies exist against which to benchmark our results. Future research to refine the model should aim to capture drivers of burnout better to improve sensitivity without compromising specificity and precision. Missing individual-level information such as their amount of and control over their workloads, workplace environment, competing demands (including personal), unique aspects of faculty roles, and working at a cancer center, as well as individual health history, limits the ability of this effort to inform burnout interventions directly and enhance their efficacy, but opens new avenues for exploration.

### Generalizability and Implications

As one of more than 70 institutions in the U.S. recognized by the U.S. National Cancer Institute with designation as a comprehensive cancer center [32], the institution from which data were analyzed for this article is a large, academic health science center focused on cancer care and research. Thus, the findings derived from this analysis may not be fully generalizable to other health care settings (e.g., non-cancer specialty or general health care focus, community hospitals, rural settings); however, the comprehensive cancer centers and other institutions that share key characteristics with our institution (e.g., focus on both research and health care delivery, university-based, large, multi-generational, racially and ethnically diverse, clinical and research faculty bodies, strong institutional engagement indicated by high survey response rate) may find these results informative for exploring drivers of burnout among their own faculty. Of course, knowledge of drivers of biomedical faculty burnout must be gathered from across multiple settings, both similar and different from our institution, to verify and refute, as well as to expand the findings we report to inform effective interventions. Regardless of setting, these results emphasize the need to implement targeted strategies against faculty burnout.

Interventions should focus on mitigating factors contributing to burnout, particularly for those previously reporting burnout, as they remain at higher risk [33]. Strategies that promote institutional inclusion and enhance support for adapting to change should be tested. Well-designed formal Human Resources systems (e.g., selection, performance management, employee and leader training, and succession planning) can play a critical role in organizational norms, culture, and performance [34]. These systems should provide an aligned and cohesive infrastructure that sets and upholds explicit expectations for competencies related to inclusion and organizational-change readiness. Beyond such systems, inclusion can be enhanced through the creation of and support for programs and activities such as employee resource groups [35]. Employee perceptions of support for change can be strengthened through the use of systematic, evidence-based frameworks of change that incorporate leadership commitment, communication, and meaningful stakeholder engagement [36]. Initiatives to improve faculty members’ sense of accomplishment and empowerment could also serve as effective buffers against burnout, providing autonomy in role design [37] and management. The impact of such policies and programs on burnout, inclusion, and change support should be measured systematically through formal mechanisms such as annual institution-wide surveys [38].

Institutional leadership also plays a vital role in shaping work environments [39]. Training leaders to foster inclusive settings, to enhance their capacity to support, and to lead change may be a critical intervention to recognize symptoms of and mitigate burnout among their team members [40]. In these ways, they can build confidence in their institutional leadership among faculty. Further, workshops focused on leadership skills, time management, and decision-making can develop these skills and foster empowerment, enabling individuals to take on challenges and influence their environments effectively and collaboratively.

## 5. Conclusions

Prior experience of burnout was the strongest factor associated with future burnout, emphasizing its state-dependent nature. However, prior burnout did not inevitably lead to a second report of burnout—those faculty who felt institutionally included and supported through changes were less likely to report burnout later, even after previous experience of burnout. Lower feelings of institutional inclusion, limited leadership support for change, and low job accomplishment increased burnout risk, while higher accomplishment and empowerment—reported at the same time as the outcome—were seemingly protective, underscoring the importance of both organizational and individual factors. Addressing burnout requires coordinated action across individual, leadership, and institutional levels, with leaders fostering inclusion and adaptability, and organizations implementing systems that enhance empowerment and well-being. Future work should examine how such factors associated with burnout evolve and test interventions that strengthen inclusion and support, guiding strategies to prevent burnout and sustain faculty well-being.

## Figures and Tables

**Table 1:** Demographic Characteristics.

Variable		Frequency	%
Sex	Female	449	40
	Male	683	60
Race	Asian	453	40
	Black or African American	41	3.6
	Hispanic, Latino or Spanish origin	79	7
	White	555	49
	Others	4	<1
Rank	Instructor	69	6
	Assistant Professor	353	31
	Associate Professor	315	28
	Full Professor	342	30
	Division Head/Chairs	53	5
Years in Institution	≤5 yrs	333	29
	6–10 yrs	288	25
	11–15 yrs	226	20
	16–20 yrs	154	14
	>20 yrs	131	12
Generation	Traditionalist-Boomer	383	34
	Gen X	624	55
	Millennial	124	11

**Table 2:** Matrix of Actual versus Predicted Faculty Burnout.

		Predicted	
		Burnout	Not Burned Out
**Actual**	**Burnout**	True positive (TP): 70 faculty	False Negative (FN): 43 faculty
	**Not Burned Out**	False positive (FP): 27 faculty	True Negative (TN): 199 faculty

**Table 3:** Variable Importance Rankings for the CART Model.

Rank	Predictor Variable	Importance Score	Relative Importance (%)
1	Burnout in 2019	89.15	100.00%
2	Job accomplishment in 2021	17.73	19.89%
3	Development in 2019	12.9	14.47%
4	Inclusion in 2021	11.52	12.93%
5	Satisfaction with manager in 2019	9.78	10.96%
6	Institutional recommendation to others in 2019	9.38	10.53%
7	Overall satisfaction with the institution in 2019	9.04	10.14%
8	Department leader effectiveness in 2019	8.99	10.09%
9	Feeling supported for change in 2021	6.56	7.35%
10	Empowerment in 2021	6.3	7.06%
11	Division leadership in 2021	5.84	6.55%
12	Leadership Alignment with Institutional Core Values in 2021	5.13	5.75%
13	Organizational integrity in 2021	4.1	4.60%
14	Years at institution	2.79	3.13%
15	Race	2.44	2.74%
16	Department leader’s support of change in 2021	2.34	2.63%
17	Manager effectiveness in 2021	2.19	2.45%
18	Generation	1.91	2.15%
19	Inclusion in 2021	1.66	1.86%
20	Feeling proud in 2021	1.61	1.81%
21	Survey effectiveness in 2021	1.47	1.65%
22	Institutional recommendation to others in 2021	1.21	1.36%
23	Alignment in 2019	1.18	1.32%
24	Executive leadership in 2021	1.13	1.27%

## Data Availability

The data supporting the findings of this study are not publicly available due to privacy and ethical restrictions. However, deidentified data may be made available by the corresponding author upon reasonable request, provided that such requests comply with applicable institutional and ethical guidelines.

## Abbreviations

The following abbreviations are used in this manuscript:

CART	Classification and Regression Tree
ANCOVA	Analysis of Covariance
MCAR	Missing Completely at Random
CP	Complexity Parameter
PPV	Positive Predictive Value

## Appendix A. Job Attitudes Categories, Reliability Coefficients, and Example Items

**Domain**	**Reliability Coefficient** **(n of Items)**		**Example Item**		**Note**
	**2019**	**2021**	**2019**	**2021**	
Agility	0.78 (2)	NA	In my work unit, change is viewed as an opportunity and not an obstacle.	NA	NA
Alignment	0.70 (3)	—^a^ (2)	I can see a clear link between my work and the institution’s goals and objectives.	I can see a clear link between my work and [the Institution Name’s] objectives.	Two items from 2021 were analyzed independently due to low correlations between them
Change Management	NA (0)	—^a^ (3)	NA	I feel supported in my efforts to adapt to changes.	New items were added in 2021
Development	0.76 (3)	NA (1)	[Institution Name] provides me with opportunities to learn new skills and develop myself.	[Institution Name] provides me with opportunities to learn new skills and develop myself.	Only one item was identical across both years, and there were more items in 2019
Job Accomplishment	NA (1)	NA (1)	My job provides me with a sense of personal accomplishment.	My job provides me with a sense of accomplishment.	Same item
Workplace Recommendation	NA (1)	NA (1)	I would recommend [Institution Name] to others as a great place to work.	I would recommend [Institution Name] to others as a great place to work.	Same item
Intention to stay	NA (1)	NA (1)	If I were offered a comparable position with similar pay and benefits at another organization, I would stay with [Institution Name].	I intend to stay with [Institution Name] at least for the next 12 months.	Almost identical item with no change in meaning
Inclusion and Diversity	0.88 (2)	0.90 (8)	[Institution Name] values and promotes diversity and inclusion at every level of the institution.	[Institution Name] values and promotes diversity, equity and inclusion at every level of the institution.	Only one item was almost identical across both years, and new items were added in 2021
Job Attitudes	—^a^ (5)	NA (0)	My job makes good use of my skills and abilities.	NA	These items were excluded in 2021
Leadership (division leader, executive leader, immediate manager)	0.85~0.92 (7)	0.86~0.92 (8)	Executive and senior leaders are taking steps to ensure [Institution Name]’s long-term success.	Executive and senior leaders are taking steps to ensure [Institution Name]’s long-term success.	Two identical items assessing executive and senior leaders were used in both years, but new items were added in 2021
Resources	NA (1)	NA (1)	I have the resources I need to successfully perform all of the things I have to do at work.	I have the resources I need to successfully perform all of the things I have to do at work.	Same item
Respect and Trust	0.89 (5)	NA (1)	I am empowered to make decisions that enable me to do my job effectively.	I am empowered to make decisions and take action within the scope of my job.	Only one item was identical across both years, and there were more items in 2019
Safety	0.79 (2)	NA (1)	I feel free to stop my work if I believe conditions are unsafe.	If a misconduct concern is raised, I am confident it will be appropriately addressed.	The same label was used in both years, but each year included different items
Patient Experience Awareness	NA (1)	NA (1)	I understand how my job affects the patient experience.	I understand how my job affects the patient experience.	Same item
Teamwork (different questions across years)	0.69 (3)	NA (0)	I can count on my coworkers to help me at work, even if they have to go out of their way to do so.	NA	Items assessing teamwork were changed in 2021, including those assessing team effectiveness and immediate manager effectiveness
My Team Effectiveness	NA (0)	0.94 (3)	NA	My team and I practice open, honest and direct communication.	
Immediate Manager	NA (0)	0.92 (3)	NA	My immediate manager recognizes my accomplishments.	
Note. —^a^ Items were analyzed as independent indicators; internal consistency is not applicable; Single item assessing belongness (2019), mentoring (2021), personal responsibility for safety (2021), institutional care (2021), survey effectiveness (2021), ethical reporting (2021) were not included in the table.					
